# Systemic sclerosis: detection of early subclinical diffuse myocardial fibrosis and impaired left ventricular strain by cardiovascular magnetic resonance

**DOI:** 10.1186/1532-429X-16-S1-O64

**Published:** 2014-01-16

**Authors:** Ntobeko A Ntusi, Stefan K Piechnik, Jane M Francis, Vanessa M Ferreira, Aitzaz B Rai, Paul M Matthews, Matthew D Robson, James Moon, Paul B Wordsworth, Stefan Neubauer, Theodoros D Karamitsos

**Affiliations:** 1 Division of Cardiovascular Medicine, Radcliffe Department of Medicine, University of Oxford & John Radcliffe Hospital, Oxford, UK; 2 GlaxoSmithKline Clinical Imaging Centre, GlaxoSmithKline, London, UK; 3 Division of Brain Sciences, Department of Medicine, Imperial College, London, UK; 4 Institute of Cardiovascular Science, University College London & Heart Hospital, London, UK; 5 NIHR Oxford Musculoskeletal Biomedical Research Unit & Nuffield Department of Orthopaedics, Rheumatology and Musculoskeletal Sciences, University of Oxford & Nuffield Orthopaedic Centre & John Radcliffe Hospital, Oxford, UK

## Background

Systemic sclerosis (SSc) is characterized by widespread tissue fibrosis including the myocardium. Diffuse myocardial fibrosis can be detected non-invasively by extracellular volume (ECV) imaging based on pre- and postcontrast T1 measurements using cardiovascular magnetic resonance (CMR). We hypothesized that multiparametric CMR, including T1 mapping, can detect subclinical myocardial involvement and provide a comprehensive cardiac assessment in patients with SSc.

## Methods

19 SSc patients (18 female, mean age 55 ± 10 years) and 20 controls (19 female, mean age 56 ± 8 years) without overt cardiovascular disease underwent CMR at 1.5T. CMR assessments included late gadolinium enhancement (LGE) [IV gadoterate meglumine at 0.15 mmol/kg], T1 mapping pre- and postcontrast, cine, tagging, and T2-weighted imaging.

## Results

Focal fibrosis on LGE was found in 10 SSc patients (53%) but none of controls. Evidence of diffuse myocardial fibrosis in SSc patients was supported by significantly higher precontrast T1 values (1007 ± 29 vs. 958 ± 20 ms, p < 0.001) and expansion of ECV (35.4 ± 4.8 vs. 27.6 ± 2.5 %, p < 0.001). Regardless of any regional fibrosis, indices of diffuse myocardial fibrosis were significantly elevated in SSc and correlated with disease activity and severity. Although biventricular size and global function were preserved, peak systolic circumferential strain (-16.8 ± 1.6 vs. -18.6 ± 1.0, p < 0.001) and peak diastolic strain rate (83 ± 26 vs. 114 ± 16 s-1, p < 0.001) were impaired in SSc. Impaired myocardial systolic strain and diastolic strain rate inversely correlated with diffuse myocardial fibrosis indices. There was no evidence of myocardial edema in SSc.

## Conclusions

Cardiac involvement is common in SSc even in the absence of cardiac symptoms, and includes both focal and ubiquitous diffuse myocardial fibrosis; this is associated with impaired systolic and diastolic strain parameters, as well as disease activity and severity. CMR may be useful in future in the study of treatments aimed at preventing or reducing diffuse myocardial fibrosis in SSc.

## Funding

This study was funded by an investigator-led grant from GSK to Dr Theo Karamitsos. The authors gratefully acknowledge support from the National Institute for Health Research Oxford Biomedical Research Centre Programme. Prof. Stefan Neubauer also acknowledges support from the Oxford British Heart Foundation Centre for Research Excellence.

**Table 1:** Continuous data are mean ± SD unless otherwise indicated.

	Controls N = 20	SSc N = 19	P value
Female sex, n (%)	19 (95)	18 (95)	0.74
Age, years	56 ± 8	55 ± 10	0.64
Hypertension, n (%)	2 (10)	4 (21)	0.41
Diabetes, n (%)	0	0	-
Hyperlipidaemia, n (%)	4 (20)	3 (16)	0.73
BMI, kg/m2	25 ± 4	27 ± 7	0.23
SSc VDAI	N/A	4 ± 2	-
ESR, mm/hr (median, IQR)	N/A	11 (3-18)	-
CRP, mg/L (median, IQR)	3 (1-4)	5 (2-8)	0.01
Hemoglobin (g/L)	13 ± 1	12 ± 1	0.05
mRSS	N/A	20 ± 6	-
LVEDV indexed, ml/m2	77 ± 16	69 ± 11	0.08
LVESV indexed, ml/m2	21 ± 5	18 ± 5	0.06
LVEF, %	73 ± 5	74 ± 6	0.52
LV Mass indexed, g/m2	52 ± 11	51 ± 8	0.74
LA size, mm	28 ± 5	37 ± 6	< 0.001
RVEDV indexed, ml/m2	85 ± 19	77 ± 12	0.32
RVESV indexed, ml/m2	28 ± 7	25 ± 7	0.06
RVEF, %	67 ± 4	67 ± 6	0.14
Mid SA circumferential strain	-18.6 ± 1.0	-16.8 ± 1.6	< 0.001
Peak diastolic circumferential strain rate (s-1)	114 ± 16	83 ± 26	< 0.001
Presence of LGE (%)	0	10 (53)	-
Volume fraction of LGE > 2SD (%)	0	3.8 ± 0.4	-
STIR T2 Ratio	1.6 ± 0.5	1.7 ± 0.4	0.66

BMI, body mass index; CRP, C-reactive protein; DMARD, disease modifying anti-rheumatic drug; ESR, erythrocyte sedimentation rate; IQR, interquartile range; LA, left atrium; LGE, late gadolinium enhancement; LV, left ventricle/ventricular; LVEDV, left ventricular end-diastolic volume; LVEF, left ventricular ejection fraction; LVESV, left ventricular end-systolic volume; mRSS, modified Rodnan skin score; RVEDV; right ventricular end-diastolic volume; RVEF, right ventricular ejection fraction; RVESD, right ventricular end-systolic volume; SA, short axis; SSc, systemic sclerosis; STIR, short Tau inversion recovery; VDAI, Valentini disease activity index of the European Scleroderma Study Group

**Figure 1:**
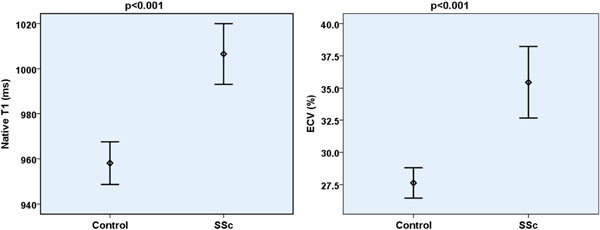
**Baseline characteristics and CMR findings**.

